# Aseptic Meningitis and White Matter Disease in Childhood-Onset Neuropsychiatric Lupus

**DOI:** 10.1155/crrh/3496303

**Published:** 2024-12-19

**Authors:** Mei Lam Hsu, Kwai Yu Winnie Chan

**Affiliations:** Department of Paediatrics, Queen Elizabeth Hospital, 30 Gascoigne Road, King's Park, Hong Kong

## Abstract

We reported a 10-year-old girl who had an atypical demyelinating disease as the presentation of her neuropsychiatric lupus. The patient had a 4-year history of systemic lupus erythematosus which had been on remission until she presented with fever and headache at the age of 10 years. Physical examination showed meningism. Extensive microbiological workup for infective meningitis was unrevealing. There was a radiographic finding of an extensive white matter hyperintensity on the magnetic resonance imaging (MRI) of the brain. At the initial stage of our case, as it was difficult to differentiate between infection of the central nervous system and neuropsychiatric manifestation of lupus, a course of intravenous immunoglobulin was given empirically instead of high-dose corticosteroid while awaiting the microbiological workup results. The fever and headache subsided shortly after commencement of intravenous immunoglobulin without use of pulse corticosteroid. After the active neurological symptoms remitted, she was given a total of six monthly doses of intravenous immunoglobulin at 2 g/kg/cycle and six biweekly doses of intravenous cyclophosphamide at 500 mg/m^2^/month. Interval MRI showed resolution of the white matter hyperintensity. Despite the extensive demyelinating disease on initial presentation, she remitted successfully without residual neurological sequelae.

## 1. Introduction

Systemic lupus erythematosus (SLE) is an autoimmune disease with different organ involvement. 15%–20% of the SLE cases were diagnosed before the 16th birthday, which are defined as juvenile-onset SLE. When compared with adult-onset SLE, juvenile-onset SLE is reported to have more different organ involvements at diagnosis and is associated with more severe organ damage [1]. Neuropsychiatric manifestation was observed in approximately one-fourth of the juvenile-onset SLE. Majority of the juvenile-onset SLE with neuropsychiatric lupus developed the neuropsychiatric events within the first 2 years after the diagnosis of SLE, among which 22% having neuropsychiatric symptoms at diagnosis of SLE and 32.8% within the first year after diagnosis [2]. The commonest neuropsychiatric manifestations in children were headache (50.3%), seizure (38.3%), and acute confusional state (33.6%) [3]. 30%–70% patients had recurrence of the neuropsychiatric events during their disease course with an average recurrence of two to three neuropsychiatric events per person. The presentation of neuropsychiatric lupus could be very variable, ranging from mild symptoms such as episodic headache, to debilitating complications like demyelinating disease or cerebrovascular disease. A multicenter study in Turkey identified antiphospholipid positivity, high SLEDAI scores, and need of plasmapheresis to be the risk factors for neurological sequelae [3]. For patients with severe neuropsychiatric symptoms, the initial first-line treatment involves use of high-dose glucocorticoids and monthly IV cyclophosphamide for 6 months, followed by maintenance therapy with oral prednisolone, azathioprine, mycophenolate, or cyclosporin. In refractory disease, rituximab and plasmapheresis are to be considered.

## 2. Case History

Our patient was 6 years old when she first complained of polyarthralgia, facial rash, and oral ulcers. Serological workup showed raised C-reactive protein (CRP) 23.3 mg/L, raised erythrocyte sedimentation rate (ESR) 50 mm/hr, and positive antinuclear antibody (ANA) 1 : 320. Anti-double-stranded deoxyribonucleic acid (anti-dsDNA) antibody at that time was 21.3 iu/mL and complement levels were normal (C3 complement = 1.33 g/L; C4 complement = 0.35 g/L). There was no renal involvement, and ophthalmological examination was normal. The SLEDAI score was 9 at the initial presentation. She does not have any family history of autoimmune disease. Oral hydroxychloroquine was started. According to the medical record, her polyarthralgia and cutaneous manifestations subsided.

**Figure 1:**
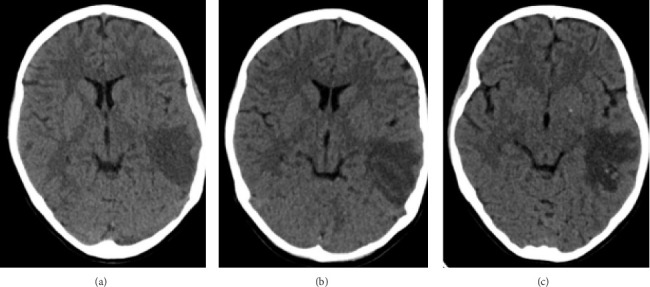
CT brain without contrast enhancement taken on Day 1 (a), Day 4 (b), and Day 10 (c) of admission, respectively. There was an extensive white matter hypodensity in the left temporal lobe on plain CT brain on admission (a) with mild enlargement on Day 4 of admission (b). There was no intracranial hemorrhage on initial presentation. The interval scan on Day 10 of admission (c) showed new hyperdensities with the hypodensity in left temporal lobe, which may be compatible with hemorrhagic component.

Four years later, when she was 10 years old, she developed a high fever of up to 39.5°C with headache and repeated vomiting. She had a normal conscious state on admission. She did not have focal neurological deficit, seizure, or personality change. Brudzinski's sign of meningitis was positive. An urgent plain computed tomography (CT) of the brain (Figure 1) showed an extensive hypodensity of undetermined nature in the left temporal lobe. There was no cerebral edema, intracranial hemorrhage, or space-occupying lesion at the first brain CT. Lumbar puncture showed an elevated opening pressure of 29 mm H_2_O with pleocytosis. The white blood cell in the CSF was raised to 485 cell/mm^3^ with predominantly polymorphs (91.7%). The CSF protein was also markedly elevated with 1526 mg/L (Ref: 150–450 mg/L). CSF glucose was 3.6 mmol/L. (Ref: 3.3–4.4 mmol/L). CSF gram stain was negative. Electroencephalogram (EEG) showed focal slowing with a sharp wave over the left posterior temporal area, suggesting a focal pathology compatible with the radiographic findings. There was no epileptiform discharge. She was empirically put on broad-spectrum antibacterial and antiviral agents for coverage of infectious meningoencephalitis, but the fever and signs of meningism persisted. Because of the uncertainty about the diagnosis, she was referred to us for further management.

Her blood test showed that CRP was high at 14.7 mg/L (Ref: < 1 mg/L), ESR was high at 78 mm/hr (Ref: 3–28 mm/hr), complement 3 (C3) was low at 0.27 g/L (Ref: 0.9–1.61 g/L), and complement 4 (C4) was low at < 2 g/L (Ref: 0.1–0.4 g/L). There were also high ANA titer 1 : 2560 and markedly raised anti-dsDNA > 300 IU/mL (Ref: < 50 IU/mL). Anti-N-methyl-D-aspartate (anti-NMDA), antineuromyelitis optica (anti-NMO), and antimyelin oligodendrocyte glycoprotein (anti-MOG) antibodies were negative. Lupus anticoagulant and anticardiolipin antibody were both negative. Extensive microbiological workups for infective meningoencephalitis with multiplex polymerase chain reaction (PCR) for meningitis panels and latex agglutination were negative for common meningitis pathogens. Specific investigations for herpes zoster virus, human polyomavirus (JC virus), Japanese encephalitis, mycobacterium tuberculosis, melioidosis, and fungal infections were negative. CSF culture was negative too.

**Figure 2:**
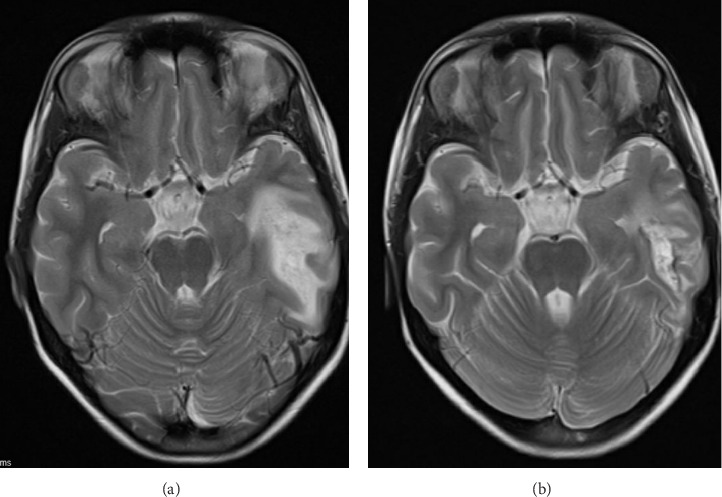
T2-weighted MRI brain taken on Day 3 and in week 4 of admission, respectively. On initial presentation (a), an area of hyperintensity was seen in the subcortical and deep white matter of left temporal lobe in T2W images. The overlying cortex in the left temporal lobe is mildly swollen with sulcal spaces effacement. The differential causes of the above radiological findings included encephalitis and demyelinating diseases. The MRI 4 weeks later (b) showed resolving T2W hyperintensity and cerebral edema after start of high dose systemic corticosteroids and intravenous immunoglobulin. However, blooming artefact was present at the posteroinferior aspect, suggestive of subacute hemorrhage product. Microhemorrhage could be a result of cerebral vasculitis. (a) On Day 3 of admission. (b) In week 4 of admission.

Magnetic resonance imaging (MRI) of the brain (Figures 2(a) and 2(b)) showed a T2W hyperintensity in the left temporal lobe's subcortical and deep white matter with mild swelling in the cortex. There were also multiple tiny foci of blooming artefacts in the susceptibility-weighted images, likely due to subacute petechial hemorrhage. Magnetic resonance arteriogram (MRA) and magnetic resonance venogram (MRV) did not reveal any aneurysm or stenosis in the internal carotid artery, circle of Willis, and its major proximal branches, while the major dural venous sinuses were patent. Magnetic resonance spectroscopy (MRS) did not demonstrate a lactate peak. The differential diagnoses from the MRI result included acute encephalitis or demyelinating disease.

Given the positive lupus serology and the 24 h urine protein of 0.54 g/day with serum albumin of 24 g/L (ref: 37–47 g/L), a renal biopsy was decided to delineate any evidence of organ involvement in active lupus. Her renal histology confirmed lupus nephritis Class IV-S (A), ISN/RPS 2003, with an activity index of 9/24 and chronicity of 0/12.

While awaiting the microbiological results, instead of giving intravenous pulse methylprednisolone, oral dexamethasone 0.5 mg/kg/day divided every 8 h was given for the first 3 days to reduce cerebral edema. The steroid dose was subsequently changed to an anti-inflammatory dose equivalent to 2 mg/kg/day of prednisolone. Intravenous immunoglobulin 400 mg/kg per day for 5 days (total 2 g/kg) was given. The total IVIG dose of 2 g was spaced out over 5 days to avoid hyperviscosity that may exacerbate thrombotic risk in cerebral vasculitis. In addition, a six biweekly dose of IV cyclophosphamide was also given at 250 mg/m^2^/dose for 3 months (i.e., 500 mg/m^2^/month for 3 months). Her fever and headache subsided shortly after the commencement of IVIG. In view of the good clinical response to IVIG, she was continued with IVIG at 2 g/kg/dose for six cycles as adjuvant therapy. She responded satisfactorily to the treatment regime with normalization of CRP, ESR, complement levels, and anti-dsDNA. Her proteinuria resolved. Serial MRIs done at 3 months, 1 year, and 2 years postevent showed interval resolution of the abnormal MRI signals at the left posterior temporal lobe. The patient's lupus disease remained in remission with a low dose of prednisolone, mycophenolate mofetil, and hydroxychloroquine. There was no residual neurological deficit resulting from the cerebral insult.

## 3. Discussion

SLE can affect both the central nervous system and the peripheral nervous system. It can lead to a wide variety of neuropsychiatric conditions, including demyelinating syndrome, cerebrovascular disease, movement disorder, myelopathy, seizure, confusion, cognitive impairment, and neuropathy to depression and psychosis. In a single-center 4-year retrospective review of juvenile-onset SLE involving 39 cases, 44% developed neuropsychiatric manifestations, among which all of them demonstrated psychiatric features, such as cognitive symptoms (82%), hallucinations (76%), depressed mood (35%), acute confused state (18%), and catatonia (12%) [4].

Multiple pathogenesis mechanisms are proposed for diverse manifestations in neuropsychiatric lupus (NPSLE) [5]. There are two major pathogeneses in NPSLE, namely, inflammatory and ischemic SLE. The former is a direct neuronal insult resulting from autoantibodies or cytokine-mediated inflammatory reactions and high permeability of the blood–brain barrier or blood–CSF barrier in inflammatory NPSLE. The latter is an ischemic or thrombotic process associated with immune complex deposition, immune-mediated vascular injury, or accelerated atherosclerosis, leading to cerebral microangiopathy, vascular occlusion, and hemorrhage.

Diagnosing NPSLE is often challenging for clinicians due to its multifarious manifestations. There is no specific biochemical marker or specific neuroimaging feature. In general, most patients with neuropsychiatric manifestations have serological evidence of active lupus, including raised autoantibodies, hypocomplementemia, and comanifestations in other systems. Some autoantibodies are found to have associations with specific neuropsychiatric symptoms [6]: aPL antibodies with ischemic stroke, vascular dementia, seizures, and transverse myelitis; antiribosomal-P antibodies with depression or psychosis; antineuronal antibodies with cognitive dysfunction and depression; antiganglioside antibodies with acute confusion, depression, and peripheral neuropathy. However, none of these antibodies is considered a definite marker of NPSLE. To diagnose NPSLE, common investigation modalities on presentation include autoantibody screening, infection screening, neuroimaging of brain, spine, and vasculatures; lumbar puncture; electroencephalography and nerve conduction studies, when clinically relevant, to look for active lupus serology and rule out other differential diagnoses.

In recent decades, neopterin level and interferon-alpha (IFN-*α*) protein levels in cerebrospinal fluid have been suggested to be helpful in guiding the diagnosis of active NPSLE. Neopterin, a proinflammatory chemical synthesized by macrophages upon stimulation with interferon-gamma, is found to have a significantly higher level in the cerebrospinal fluid of NPSLE patients than the SLE patients without neuropsychiatric involvement. The neopterin level and IFN-*α* protein levels in cerebrospinal fluid of NPSLE patients were both suppressed after the neuropsychiatric diseases are under treatment with immunosuppressant [7]. With further studies in proving the association of neopterin and IFN-*α* levels in NPSLE, it is hoped that these two biomarkers could more specifically guide the diagnosis of active NPSLE and thus earlier targeted treatment could be provided.

In addition to biochemical markers, with the advancement in neuroimaging technology, MRI is well recognized as an essential imaging modality for approaching the diagnosis of neuropsychiatric lupus and excluding its mimicries [8]. The main MRI features are nonspecific T2/FLAIR white matter hyperintensities (WMHs) and cerebral atrophy [4]. In a single-center study in London involving 27 pediatric NPSLE patients, abnormalities in brain MRI were reported in 41% of the NPSLE cases. Among those with abnormal MRI, the commonest finding was hyperintensity on T2W sequence (81.8%), followed by brain atrophy (45%), cortical gray matter lesions (0.9%), and basilar artery infarction (0.9%) [9]. The WMHs in brain MRI of NPSLE patients are preferentially present in the periventricular region, i.e., within a 3 mm distance from the lateral ventricles, but could also be multifocal involving the deep white matter as well [10]. The location and pattern of the WMH on MRI may aid the differentiation between inflammatory NPSLE and ischemic NPSLE. Previous studies have found that inflammatory NPSLE usually has a high volume and number of WMI in deep white matter, and the WMI has a more complex shape with a high concavity. The volume of the WMH on MRI was also reported to have an inverse relationship with the psychomotor function of the NPSLE patients [11], which poses a prognostic implication.

Given the MRI findings in our case, an important differential diagnosis was multiple sclerosis (MS). The diagnosis of MS could not be substantiated at the time of presentation as it has to demonstrate dissemination in time and place. IgG oligoclonal band in cerebrospinal fluid is an important and sensitive hallmark for MS because it is present in > 95% of patients with MS. Some radiological features may be helpful in differentiating the diagnosis between NPSLE and MS [12]. The lesions in NPSLE are not confined to periventricular areas but also the gray–white juncture at the subcortical areas or even the cortical regions. On the contrary, the lesions in MS are usually located asymmetrically along the white matter tracts in the periventricular region and may involve the corpus callosum with small ovoid lesions. Another differentiating feature between the two is the involvement of bilateral basal ganglia with swelling and punctate enhancement in NPSLE, which is uncommon in MS. T1W images may also help differentiate the two because the white matter lesions in SLE usually appear normal on T1W, whereas the demyelinating plaques in MS may be seen as hypointense areas on T1W images. These two disease entities both may involve the spinal cord with T2 high signal abnormality on the MRI spine. While lesions in MS are short, involving < 3 vertebral segments, the spinal cord lesions in SLE are often longitudinally extensive [13].

Apart from MS, another possible differential diagnosis for the white matter lesions on MRI included progressive multifocal leukoencephalopathy (PML), typically leading to multifocal areas of white matter demyelination. We had tested for JC virus in our patient as it is a known cause of PML in immunocompromised hosts, and JC virus was not detected in the CSF of our patient. Posterior reversible leukoencephalopathy syndrome (PRES) was also another differential diagnosis to be considered but is less likely in this case as the hyperintensities on T2W imaging in PRES are typically found in the parietal and occipital lobes. Also, our patient did not have hypertension, a common predisposing event in PRES, and did not have any visual symptoms on presentation.

Another spectrum of manifestations in NPSLE is cerebral vascular disease due to secondary vasculitis, or thrombophilia in secondary antiphospholipid syndrome [14]. SLE vasculopathy predominantly affects arterioles and capillaries resulting in lacunar infarcts and large cerebral infarcts. Apart from arterial thrombosis, there is also an increased tendency of venous sinus thrombosis in SLE, giving rise to generalized cerebral edema and sometimes cortical venous infarction with hemorrhagic foci. MRV plays a key role in confirming the diagnosis. All these were negative in our patient, making ischemic NPSLE less likely than inflammatory NPSLE.

In addition to MRI, brain perfusion imaging with single-photon emission computerized tomography (SPECT) is another imaging modality that has been of great clinical interest in the past two decades for the diagnosis of NPSLE. With the capability of detecting a local change in cerebral blood flow shortly after a brain insult, SPECT is highly sensitive in detecting vasculitic processes or cerebral inflammatory reactions in over 90% of NPSLE patients [15]. Although SPECT alone lacks specificity in diagnosing NPSLE, it can potentially detect early disease when used as an adjunct to other clinical assessments and investigations and thus facilitate early treatment.

In our patent, the presence of neck rigidity raised intracranial pressure and CSF pleocytosis raised a significant concern on CNS infection. With more investigation results available, her positive lupus serology and evidence of concomitant lupus nephritis on renal biopsy helped us to make the diagnosis of demyelinating disease of NPSLE with escalation of immunosuppressive therapy.

Some centers also reported diagnostic challenges of NPSLE as in our case. Ferraria et al. in Department of Pediatrics, Hospital Nossa Senhora do Rosário, reported a case of a 7-year-old girl presenting with acute onset of ataxia, diplopia, morning vomiting, and severe headache. The brain CT showed nonspecific hypodensity in the cerebellum while the brain MRI showed multiple hyperintense signals on T2W sequence in both temporal lobes, right insula, right anterior superior frontal lobe, and left frontal lobe. Given the clinical and radiological features, encephalitis was suspected. Their initial treatments were ceftriaxone, acyclovir, and tuberculostatics to empirically cover infective meningitis. Dexamethasone was also started for possible diagnosis of acute demyelinating encephalomyelitis. Her serological test results later revealed a high antinuclear antibody titer (1 : 5120) and positive anti-ds DNA antibodies. After the diagnosis of NPSLE was established, immunosuppressive treatment was then escalated. She received cyclophosphamide and monthly pulses of methylprednisolone for a 6-month induction, followed by oral prednisolone as maintenance therapy. She had marked clinical response after first dose of cyclophosphamide and methylprednisolone [16].

As NPSLE carries high morbidity and mortality, prompt diagnosis and initiation of treatment are important in hastening disease progression. In the acute treatment of NPSLE, the first line of treatment consists of high-dose glucocorticoids of 30 mg/kg/day for three consecutive days, followed by an equivalent dose of prednisolone 1–2 mg/kg/day tapering over 3–12 months, plus six monthly doses of intravenous cyclophosphamide 0.5–1 g/m^2^/month [17]. However, the diagnosis of NPSLE is often complicated and requires extensive workup to rule out other possible diagnoses. Early and intensive immunosuppressive treatment is crucial, but we have to balance the risk while waiting for the microbiological and other investigation results. We thus started the patient on a low-dose cyclophosphamide at 0.25 g/m^2^/dose two weekly for 6 doses with monthly IVIG as adjuvant therapy. This dose of IV cyclophosphamide was low compared to the usual standard. The disease, however, responded exceptionally well and went into remission, as evidenced by the defervescence, normalization of biochemical markers, and resolution of neuroimaging abnormalities. Hence, this low-dose cyclophosphamide was continued without escalation of dosage.

## 4. Conclusion

It is a challenging task to differentiate a CNS infection from other CNS involvement in SLE. Cerebral lupus can present with clinical features of aseptic meningitis and white matter disease of the brain. The degree of involvement may be out of proportion to the clinical presentation. Low-dose intravenous cyclophosphamide with monthly IVIG can be a treatment option for selected NPSLE patients.

## Data Availability

This is a case report and does not link to any data.
